# IgG glycopeptide enrichment using hydrophilic interaction chromatography-based solid-phase extraction on an aminopropyl column

**DOI:** 10.1007/s00216-024-05187-y

**Published:** 2024-02-13

**Authors:** Katarina Molnarova, Michaela Chobotova, Petr Kozlik

**Affiliations:** Department of Analytical Chemistry, Faculty of Science, Charles University, Hlavova 8, 128 43 Prague 2, Czech Republic

**Keywords:** Glycoproteomics, Glycopeptide enrichment, Solid-phase extraction, Hydrophilic interaction liquid chromatography, Immunoglobulin G

## Abstract

The sample preparation step is pivotal in glycoproteomic analysis. An effective approach in glycoprotein sample preparation involves enriching glycopeptides by solid-phase extraction (SPE) using polar stationary phases in hydrophilic interaction liquid chromatography (HILIC) mode. The aim of this work is to show how different experimental conditions influence the enrichment efficiency of glycopeptides from human immunoglobulin G (IgG) on an aminopropyl-modified SPE column. Different compositions of the elution solvent (acetonitrile, methanol, and isopropanol), along with varying concentrations of elution solvent acidifiers (formic and acetic acid), and different concentrations of acetonitrile for the conditioning and washing solvents (65%, 75%, and 85% acetonitrile) were tested to observe their effects on the glycopeptide enrichment process. Isopropanol proved less effective in enriching glycopeptides, while acetonitrile was the most efficient, with methanol in between. Higher formic acid concentrations in the elution solvent weakened the ionic interactions, particularly with sialylated glycopeptides. Substituting formic acid with acetic acid led to earlier elution of more glycopeptides. The acetonitrile concentration in conditioning and washing solutions played a key role; at 65% acetonitrile, glycopeptides were not retained on the SPE column and were detected in the flow-through fraction. Ultimately, it was proven that the enrichment method was applicable to human plasma samples, resulting in a significant decrease in the abundances of non-glycosylated peptides. To the best of our knowledge, this study represents the first systematic investigation into the impact of the mobile phase on glycopeptide enrichment using an aminopropyl-modified SPE column in HILIC mode. This study demonstrates the substantial impact of even minor variations in experimental conditions, which have not yet been considered in the literature, on SPE-HILIC glycopeptide enrichment. Consequently, meticulous optimization of these conditions is imperative to enhance the specificity and selectivity of glycoproteomic analysis, ensuring accurate and reliable quantification.

**Supplementary Information:**

The online version contains supplementary material available at 10.1007/s00216-024-05187-y.

## Introduction

Protein glycosylation is a result of complex, enzymatically controlled processes during which units of sugars, also known as glycans, are covalently attached to the protein residue [1]. Glycoproteins are essential macromolecules, which perform many important functions such as lubrication and protection, intracellular migration, sorting and secretion, antigen–antibody interaction, signal transduction, virus–cell interaction, and many others [2]. It has been proved that the alteration in glycosylation can be used as specific biomarkers for cancer [3], Alzheimer’s diseases [4], or diabetes [5]. Moreover, in the last decade, biotherapeutic products based on glycoproteins have been introduced to the pharmaceutical industry. Since glycosylation influences the stability, activity, pharmacokinetics, and pharmacodynamics of these drugs, it is essential to control their development process [6]. In bottom up, also called shotgun (glyco)proteomics, (glyco)proteins are extracted from cell or tissues and then digested to smaller (glyco)peptides by proteolysis (usually by trypsin) prior to their separation by liquid chromatography coupled to high-resolution mass spectrometry [7]. The complexity of both the peptide sequence and glycan composition makes their analysis challenging, despite recent progress in bioanalytical methods [8]. The challenges in glycopeptide analysis primarily arise from their lower ionization efficiency compared to non-glycosylated peptides, as well as their low concentration levels in complex mixtures such as blood and other body fluids [9]. In bioanalytical analysis, the most common issues in glycoprotein and glycopeptide analysis include the presence of a large number of proteins, peptides, electrolytes, and various other small components. All of these factors complicate the analysis of body fluids as a potential source of disease biomarkers [10].

To overcome these limitations, various enrichment techniques have been devised. Enrichment methods can be classified into several groups, with the most prominent ones involving lectin affinity enrichment [11], hydrazide-based approaches [12], boronic acid chemistry [13], and immunoprecipitation methods [14]. While these enrichment techniques have proven effective, they come with certain limitations. Lectin affinity methods, for instance, are limited by the fact that a specific lectin can only bind to a single glycan structure [15]. On the other hand, techniques relying on covalent interactions (such as hydrazide and boronic acid chemistry) solely provide information about the glycosylation site; they do not allow determination of the glycan structure or degree of site occupancy. Additionally, these methods lack specificity, as non-glycosylated peptides can interfere with the enrichment process. Moreover, they entail intricate reactions and are time-consuming [16].

An alternative approach for glycopeptide enrichment involves SPE on polar stationary phases in HILIC mode. In HILIC, the enrichment of the glycopeptides is based on partitioning of the polar analyte (represented by the attached glycan moiety) between the water-enriched polar stationary phase (bare or modified silica) and mobile phase with high concentration of organic solvent [17]. The primary advantage of HILIC-based glycopeptide enrichment lies in its specificity toward glycosylated peptides. On the polar stationary phase, glycopeptides are preferentially retained over non-glycosylated tryptic peptides. Several studies have employed HILIC-based enrichment methods in their researches. Zacharias used a modified cotton wool for the enrichment of glycopeptides derived from breast and brain cancer cells [18]. Cao’s research team employed a zwitterionic functionalized soluble nanopolymer for the enrichment of both glycopeptide standards and glycoproteins of human serum [19]. Yang et al. focused on enriching *N-* and *O*-linked glycopeptides from human breast cancer tumor tissues using a sulfobetaine type of zwitterionic stationary phase (ZIC-HILIC) [20]. However, none of these studies explored the impact of various experimental conditions on the enrichment process.

To our knowledge, only one study has compared the efficiency and selectivity of glycopeptide enrichment using different solvents on ZIC-HILIC. This research examined acetonitrile, methanol, ethanol, and isopropanol for enriching IgG and α1-antitrypsin standards from human plasma. The study found that the choice of organic solvent significantly influenced the enrichment efficiency, primarily based on the peptide backbone hydrophobicity. Isopropanol and ethanol favored more hydrophobic glycopeptides, whereas acetonitrile, despite co-enriching fewer peptides, proved the best solvent for both hydrophilic and hydrophobic glycopeptides. Methanol, however, performed poorly likely due to its competition with water uptake from the mobile phase, leading to insufficient retention of polar analytes [21]. This study primary explored solvent effects on glycopeptide enrichment using ZIC-HILIC, yet the results underscore the broader impact of experimental variables from sample choice to solvent composition on enrichment efficiency. Based on this study, it is obvious that the experimental conditions in HILIC-based SPE play crucial role in the efficiency and selectivity of glycopeptide enrichment. Therefore, there is a high demand to further investigate the possibilities of this enrichment method in glycopeptide analysis. We present here a comprehensive study exploring various experimental conditions employed for enriching human IgG glycopeptides on aminopropyl-modified SPE column in HILIC mode. This research thoroughly investigates the experimental conditions that impact the efficiency of glycopeptide enrichment in HILIC-SPE, an aspect that has been explored to a limited extent in previous studies. We examined in detail the outcomes of enrichment on the aminopropyl-modified sorbent using different types of organic elution solvents (acetonitrile, methanol, isopropanol), varied concentrations and types of elution solvent acidifiers, and the concentration of acetonitrile in the elution, conditioning, and washing solvents. Our study demonstrates that altering experimental parameters, specifically varying concentrations and types of elution solvent acidifiers and acetonitrile concentration in conditioning and washing solvents, factors not yet considered in the literature, can effectively influence the extraction efficiency of individual glycopeptides. The obtained findings are useful for selecting the appropriate solvents and optimal experimental conditions for the HILIC-SPE glycopeptide enrichment approach, ensuring efficient and accurate enrichment of glycopeptides. Therefore, these findings are valuable for scientists working in glycoproteomic analysis.

## Materials

The following chemicals were purchased from Sigma-Aldrich (St. Louis, MO, USA): standard of human IgG (p/n I4506), iodoacetamide (purity ≥ 99%), dithiothreitol (purity ≥ 99%), SOLu-Trypsin (p/n EMS0004), acetonitrile, methanol, and isopropanol (each LC-MS grade). Water and ammonium hydrogen carbonate (both LC-MS grade) were supplied by Merck (Darmstadt, Germany). Formic acid (LC-MS grade) was supplied by Thermo Fisher Scientific (Waltham, MA, USA) and acetic acid (LC-MS grade) from Avantor (Radnor, PA, USA). The SPE column filled with aminopropyl-modified silica gel (45 µm; 100 mg sorbent/1 mL, 60 Å) used for the enrichment was provided by Macherey-Nagel (Düren, Germany). Plasma of healthy volunteers was provided by Dr. Lenka Hasikova (First Faculty of Medicine, Charles University, Prague, Czech Republic). All volunteers provided their written consent regarding biological sample collection and storage prior to participation. Samples were collected in accordance with standards set by the institutional ethics committee (no. 6181/2015).

### Sample preparation and enrichment

**Fig. 1:**
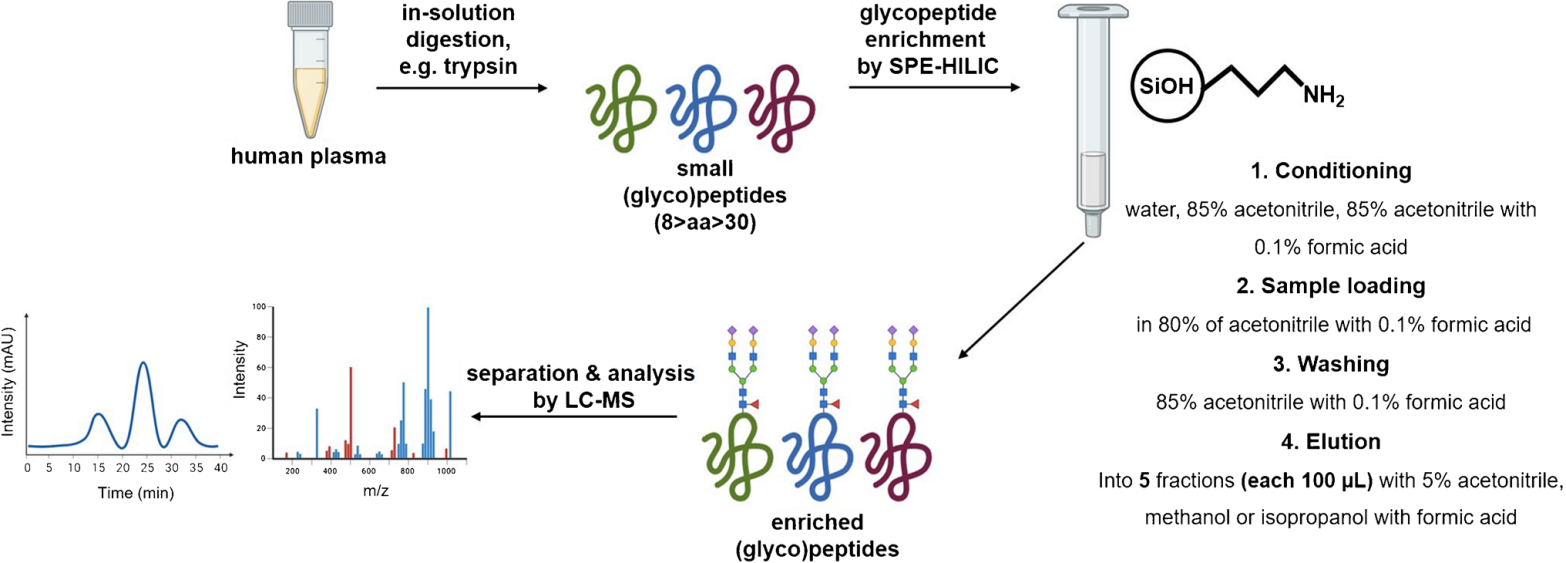
Workflow of the sample preparation and enrichment process

The enrichment was performed on a tryptic digest of human IgG. For the digestion, 300 µL (4 µg µL^−1^) of the IgG standard dissolved in ammonium hydrogen carbonate was used, and the sample was prepared according to a previously published protocol [22]. Briefly, IgG glycopeptide standard was prepared by tryptic digest of 300 µL of IgG solution (4 µg µL^−1^ dissolved in 50 mM ammonium hydrogen carbonate). Cysteine residues were reduced with 5 mM dithiothreitol (60 min at 60 °C), alkylated with 15 mM iodoacetamide (30 min in the dark), and residual iodoacetamide was quenched with 5 mM dithiothreitol. Trypsin was added to the mixture at an enzyme/protein ratio 1:40 (w/w) and allowed to undergo digestion (overnight at 37 °C), and the next day, trypsin was heat-inactivated (10 min at 85 °C). Twenty microliter of IgG tryptic digest was diluted in 80 µL of a solution containing 80% acetonitrile with 0.1% formic acid. The enrichment was carried out in four steps on the aminopropyl-modified sorbent: (1) equilibration of the SPE column, washing with 1 mL water, 1 mL 85% acetonitrile (if not stated otherwise), and 1 mL 85% acetonitrile with 0.1% formic acid (if not stated otherwise); (2) loading of the sample in 80% of acetonitrile with 0.1% of formic acid, and the flow-through fraction was reapplied onto the column; (3) washing the SPE column was with 1 mL 85% acetonitrile with 0.1% formic acid (if not stated otherwise); and (4) elution of the analytes from the SPE column by 100 µL of 5% acetonitrile with 0.1% formic acid into five fractions (each fraction of 100 µL). The workflow of the sample preparation is depicted in Fig. 1. The elution of the glycopeptides into five individual fractions allowed us to investigate the enrichment of specific glycoforms in depth. Each enrichment process was carried out in triplicate, and each sample was measured three times. The efficiency of the enrichment process was assessed by comparing the relative areas of specific glycoforms. The relative area of specific glycoform was calculated as the ratio of the absolute area of a particular glycoform divided by the total area of that glycoform found in the fractions.

### Chromatographic and mass spectrometric conditions

The experiments were performed on an Agilent 1290 Infinity II LC System with a binary pump (Agilent Technologies, Inc., Waldbronn, Germany) interfaced with maXis™ Q-TOF mass spectrometer (Bruker Daltonics, Bremen, Germany). The prepared samples were separated on an ethylene bridged hybrid (BEH) C18 column (2.1 × 100 mm; 1.7 μm; p/n 186002352) supplied by Waters Corporation (Milford, MA, USA). The mobile phase consisted of water with 0.1% formic acid (Solvent A) and acetonitrile with 0.1% formic acid (solvent B). The samples were injected in 2 µL volume, the flowrate was set at 0.25 mL mi^—1^, and the column temperature was maintained at 40 °C. For the separation, the following gradient program (min/solvent B (%)) was used: 0/5–5/5–15/25–30/80–33/80–35/5–40/5. The glycopeptides were identified manually based on precursor ions and their fragmentation spectra, which were acquired in an information-dependent mode. For the acquisition, otofControl 4.0 and Hystar 3.2 software were used, while for the data evaluation, Bruker DataAnalysis 4.4 software (Bruker Daltonics, Bremen, Germany) was used. The parameters of the mass spectrometric method were set based on the previously published study [22]. All the studied glycopeptides are summarized in Table 1. The chromatograms were created by exporting and overlaying the extracted ion chromatograms of the glycopeptides to OriginPro 8.5.0 (OriginLab Corporation, Northampton, MA, USA).

**Table 1:**
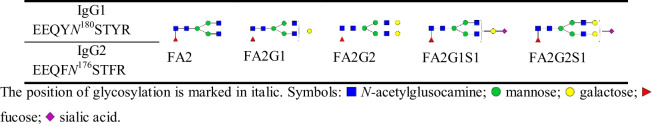
The studied biantennary glycopeptides of human IgG

## Results and discussion

**Fig. 2:**
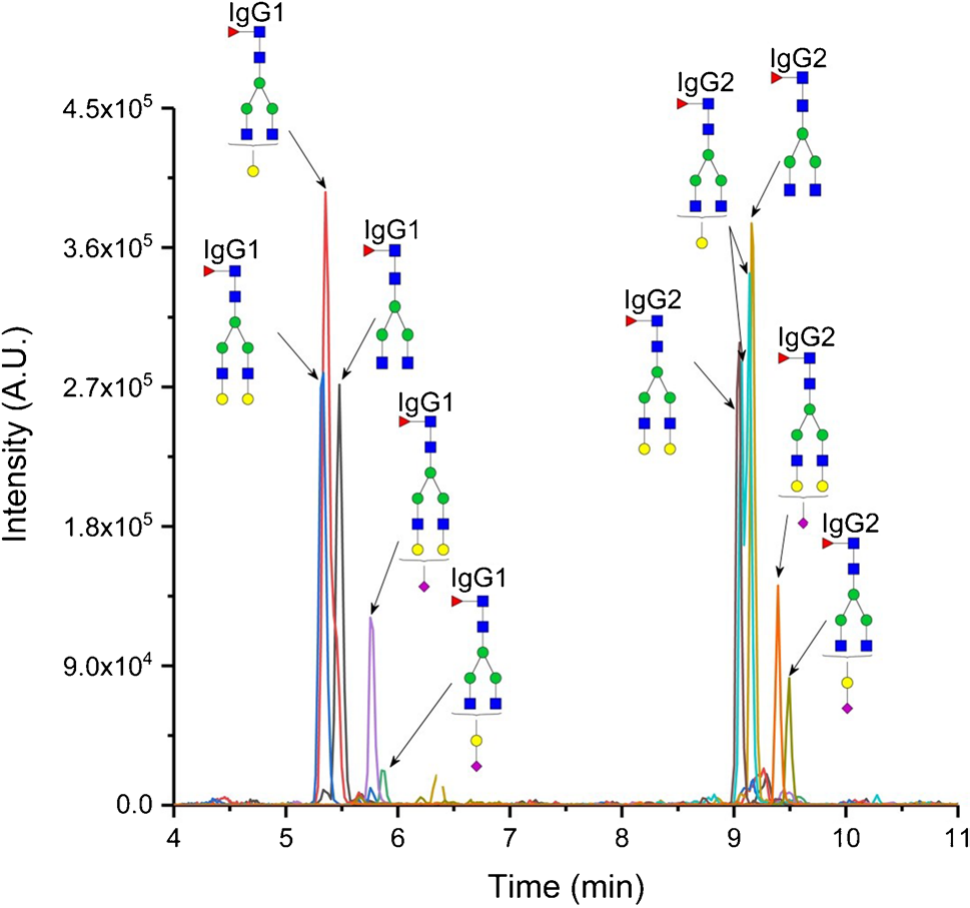
Separation of the human IgG glycopeptides after the enrichment on the aminopropyl-modified silica gel sorbent in HILIC mode. The enrichment was carried out as described in the “Sample preparation and enrichment” section

In this study, we investigated the enrichment efficiency of the biantennary *N*-glycopeptides of human IgG using the SPE-HILIC mode prior to their analysis by RP-LC/MSMS. Specifically, we focused on two tryptic glycopeptides from IgG1 and IgG2, with the peptide sequences EEQY*N*^180^STYR and EEQF*N*^176^STFR, respectively. Figure 1 illustrates the chromatogram depicting the separation of the IgG glycopeptides following enrichment on the aminopropyl-modified sorbent. For enrichment, 20 µL of the tryptic digest, corresponding to 50 µg of the IgG standard, was used, and the enrichment procedure was carried out as described in the “Sample preparation and enrichment” section. Subsequently, the five fractions (each fraction of 100 µL) after HILIC-SPE were mixed, evaporated, and reconstituted in 100 µL 5% acetonitrile with 0.1% formic acid. The separation was performed in RP-LC mode because the elution solvent used in HILIC-SPE (with a high aqueous component) is compatible with RP-LC. This allows the sample solvent to be directly injected into RP-LC without adversely affecting peak shapes. Conversely, using such a sample solvent would negatively impact HILIC chromatography, resulting in deteriorated peak shapes. As a result, if we had chosen HILIC separation, it would have necessitated evaporation of the sample after HILIC-SPE and reconstitution in a high percentage of acetonitrile. However, this procedure could potentially introduce additional errors in the glycopeptide determination process [23]. Moreover, RP-LC is more suitable for comparing the effects of the SPE method on both glycosylated and non-glycosylated peptides (chapter “Enrichment of the tryptic-digest of human plasma”), given that the studied non-glycosylated peptides would exhibit very low retention in HILIC mode. During the RP-LC separation of the IgG glycopeptides, we observed consistent glycopeptide retention trends as described in our previous study [22]. As shown in Fig. 2, the glycopeptides of IgG1 eluted in a cluster with a retention window from 5.3 to 5.9 min, while for IgG2 glycopeptides, this window was from 9.0 min to 9.5 min. In addition, the monogalactosylated glycopeptide (FA2G1) of IgG2 exhibited a partial separation of isoforms. These isoforms differ based on whether the galactose is attached to the α3 or α6 branch. In this paper, we designated the first eluting isomer as isomer 1, and the later eluting isomer as isomer 2.

### General trend of glycopeptide enrichment on the aminopropyl-modified stationary phase

**Fig. 3:**
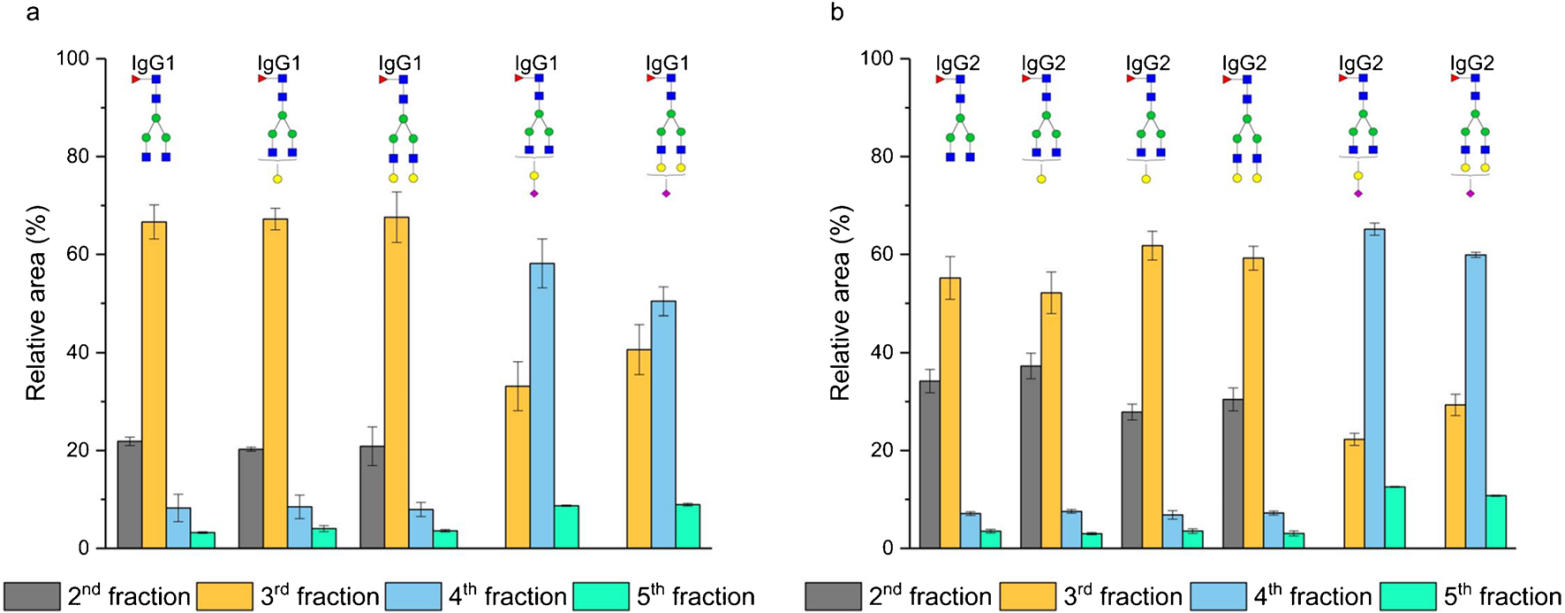
Enrichment of the IgG1 (**a**) and IgG2 (**b**) glycopeptides on the aminopropyl-modified silica gel column. The enrichment was carried out as described in the “Sample preparation and enrichment” section

In our experiment, we initially tested 5% acetonitrile with the addition of 0.1% formic acid as the elution solvent. Figure 3a illustrates that neutral glycopeptides of IgG1 gradually eluted from the second to the fifth fraction, with the majority concentrated in the third fraction (no glycopeptides were detected in the first fraction). In HILIC mode, the primary retention mechanism for neutral glycopeptides involves partitioning the polar analyte (represented by the attached glycan moiety) between the water-enriched polar stationary phase and the acetonitrile-rich mobile phase. As depicted in Fig. 3a and b, the number of galactoses did not significantly impact the enrichment process. The relative abundance of a-, mono-, and digalactosylated glycopeptides remained similar in each fraction. On the other hand, sialylated glycopeptides exhibited a stronger interaction with the stationary phase, leading to their later elution from the SPE column, predominantly in the fourth fraction. This later elution can be attributed to ionic interaction between the positively charged amino group of the stationary phase and the partly dissociated residues of sialic acid. Furthermore, our observations revealed that the peptide backbone influenced the glycopeptide retention. When comparing the enrichment of IgG1 with IgG2, we noted that glycopeptides with a more hydrophobic peptide backbone (IgG2) were less retained on the polar stationary phase, causing a higher proportion of them to elute in the earlier fractions. The peptide backbone of IgG1 and IgG2 differs in only two amino acids. The glycopeptides from IgG1 are more polar, attributed to the two tyrosines (EEQY*N*^180^STYR) in the peptide backbone. On the other hand, the glycopeptides from IgG2 are more hydrophobic due to the presence of two phenylalanines (EEQFN^176^STFR) in the peptide backbone [22].

### Effect of the organic type of the elution solvent on glycopeptide enrichment

**Fig. 4:**
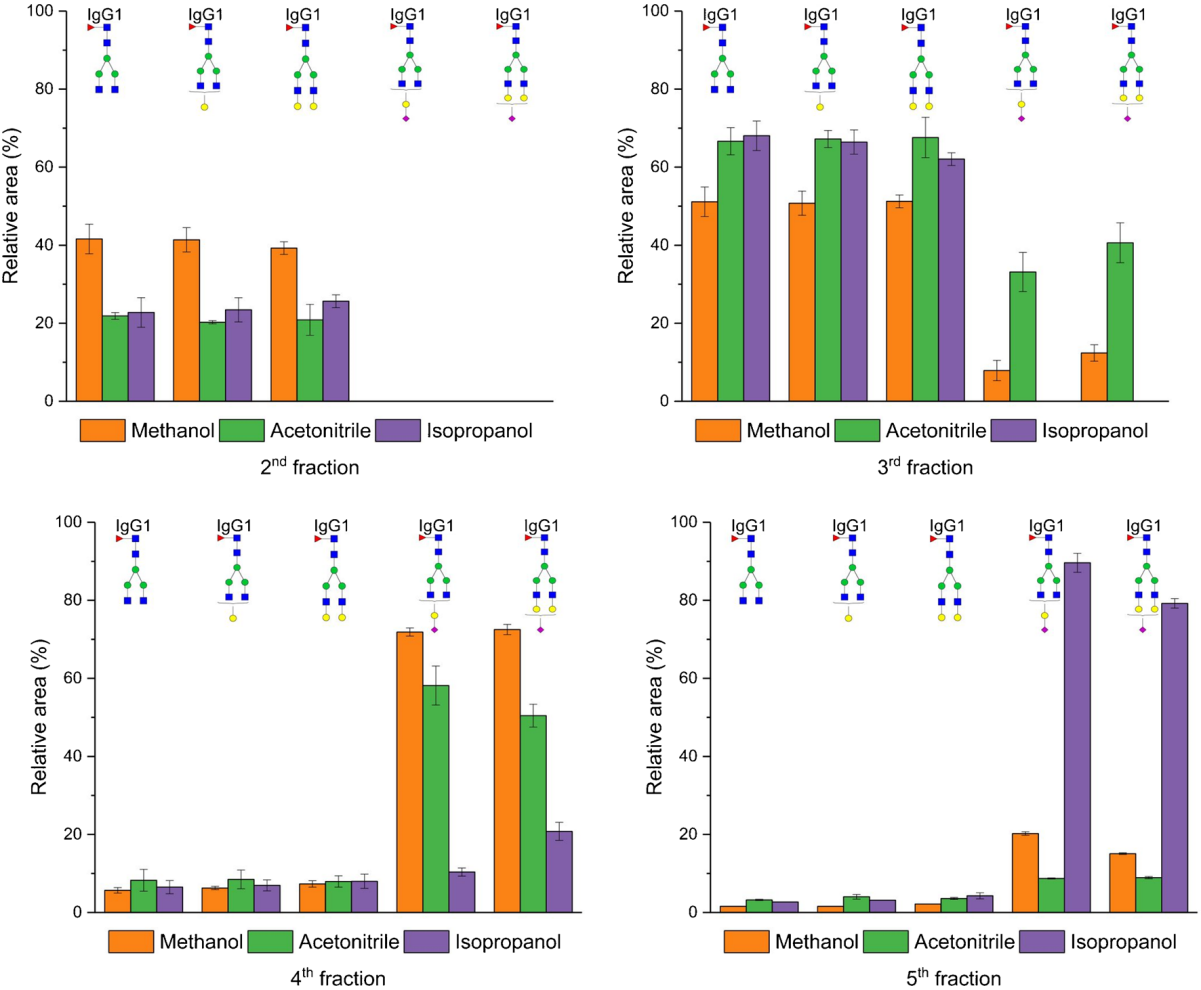
Effect of different organic modifiers of the elution solvent on the efficiency of glycopeptide enrichment

Following the initial experiment, we studied the effect of different elution solvents on the glycopeptide enrichment efficiency. In HILIC, acetonitrile, methanol, and isopropanol are commonly used as elution solvents, with their elution strength ranking as methanol > acetonitrile > isopropanol [24]. In each instance, the concentration of the organic solvent was 5% with addition of 0.1% formic acid. Figure 4 shows that methanol proved to be the most effective solvent for washing out neutral glycopeptides from the column, aligning with the HILIC eluotropic row. On the other hand, isopropanol exhibited slightly stronger elution capabilities than acetonitrile. Interestingly, none of the tested solvents displayed sufficient strength to elute sialylated glycopeptides in the second fraction. Sialylated glycopeptides only began to elute from the SPE column in the third fraction, primarily with methanol and acetonitrile, where acetonitrile exhibited higher elution efficiency over methanol. When isopropanol was employed, sialylated glycopeptides were predominantly detected in the last fraction. A similar trend was observed for IgG2 glycopeptides, where methanol emerged as the most effective solvent for eluting neutral glycopeptides, followed by acetonitrile and isopropanol (Fig. S1 in Electronic Supplementary Material). Since acetonitrile was the best elution solvent for both neutral and sialylated glycopeptides and is the most common organic solvent used in HILIC [21–25], it was chosen as the organic solvent for the further experiments.

### Effect of the formic acid concentration of the elution solvent on glycopeptide enrichment

**Fig. 5:**
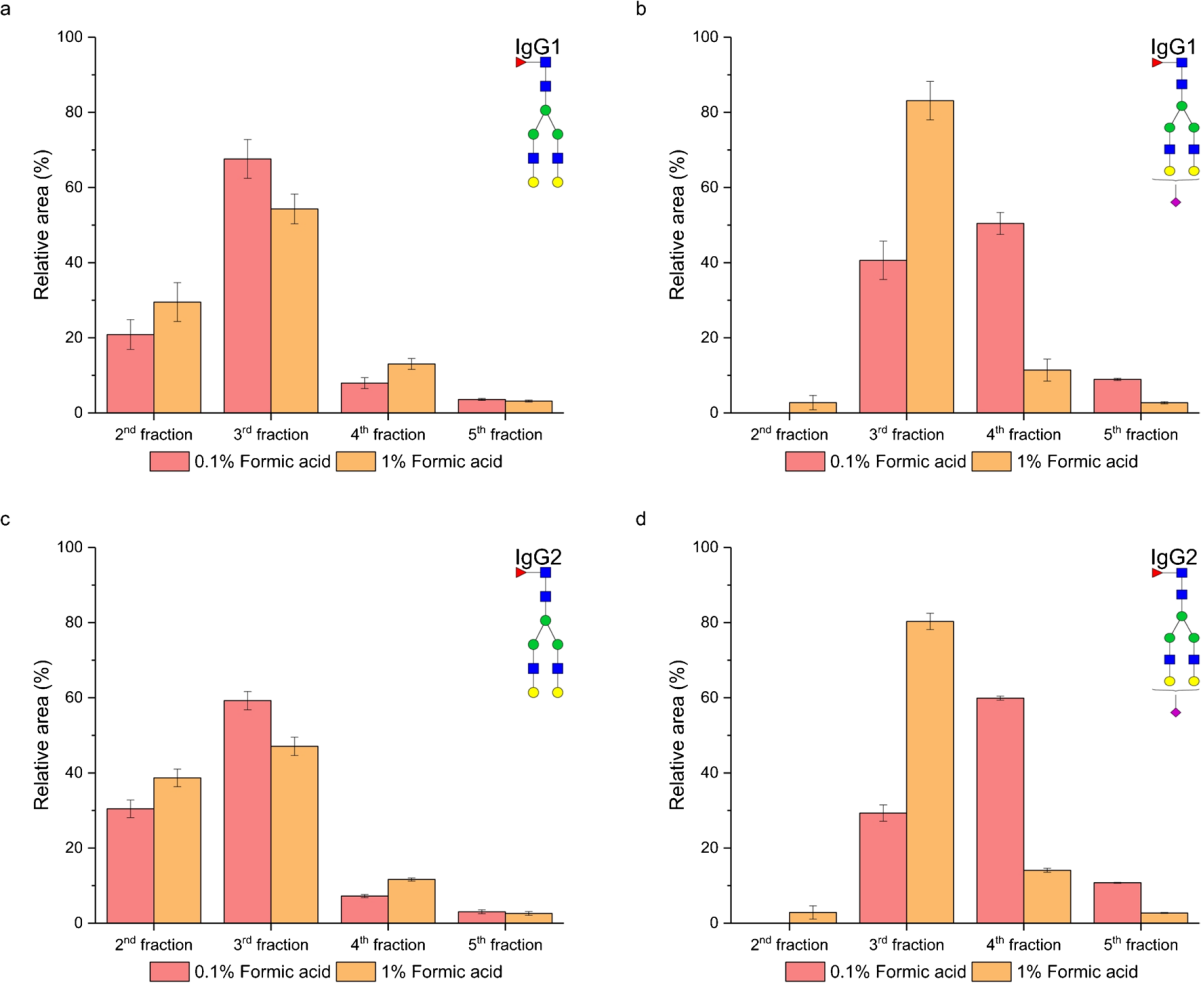
Effect of the formic acid concentration in the elution solvent on the enrichment efficiency of neutral FA2G2 glycopeptide of IgG1 (**a**) and IgG2 (**c**) and sialylated FA2G2S1 of IgG1 (**b**) and IgG2 (**d**)

We studied the effect of formic acid concentration on the enrichment efficiency. The formic acid concentration in the elution solvent was increased from the initial 0.1 to 1%, while the acetonitrile concentration remained consistent with the previous experiments. In Fig. 5a and c, it is seen that both the tested formic acid concentrations exhibited similar trends in eluting neutral glycopeptides. However, the solvent with 1% formic acid demonstrated slightly higher elution strength, leading to a higher relative abundance of these glycoforms in the second fraction.

In contrast, the sialylated glycopeptide FA2G2S1 (Fig. 5b and d), for both peptide backbones, was detected in the second fraction when the higher formic acid concentration was used. This phenomenon can be explained by the suppression of ionic interaction between the positively charged aminopropyl functional group of the stationary phase and the partly dissociated sialylated glycopeptides. This effect is more pronounced with the higher concentration of formic acid due to the elevated ionic strength of the elution solvent. The ionic strength of the elution solvents, calculated using the Peak Master program (https://web.natur.cuni.cz/gas/peakmaster.html), was determined to be *I* = 2.18 mM (0.1% formic acid) and *I* = 7.37 mM (1% formic acid), respectively. The higher ionic strength more effectively suppresses ionic interactions, causing sialylated glycopeptides to elute earlier. Another factor contributing to the earlier elution of sialylated glycopeptides is the lower pH of the more concentrated acid (pH 2.17 of 1% formic acid compared to pH 2.68 of 0.1% formic acid). This lower pH further suppresses the dissociation of sialic acid (p*K*_a_ = 2.6 [26]), rendering this glycoform more neutral and resulting in earlier elution. Overall, the elution solvent of 5% acetonitrile with 1% formic acid effectively washed out more glycopeptides in the earlier fraction. These trends were consistent across all studied neutral and sialylated glycopeptides (Fig. S2 in Electronic Supplementary Material).

### Effect of the acidifier type of the elution solvent on glycopeptide enrichment

**Fig. 6:**
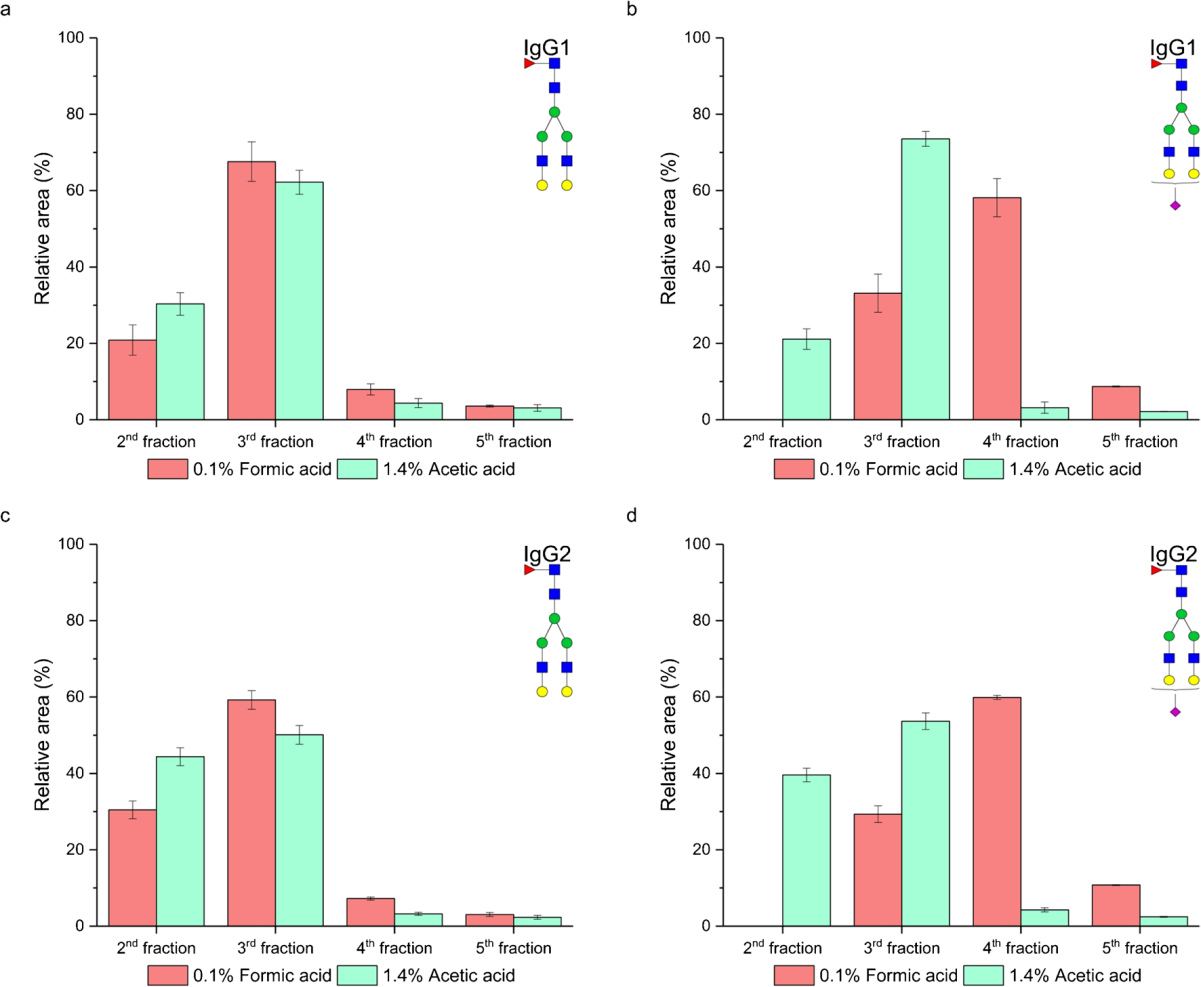

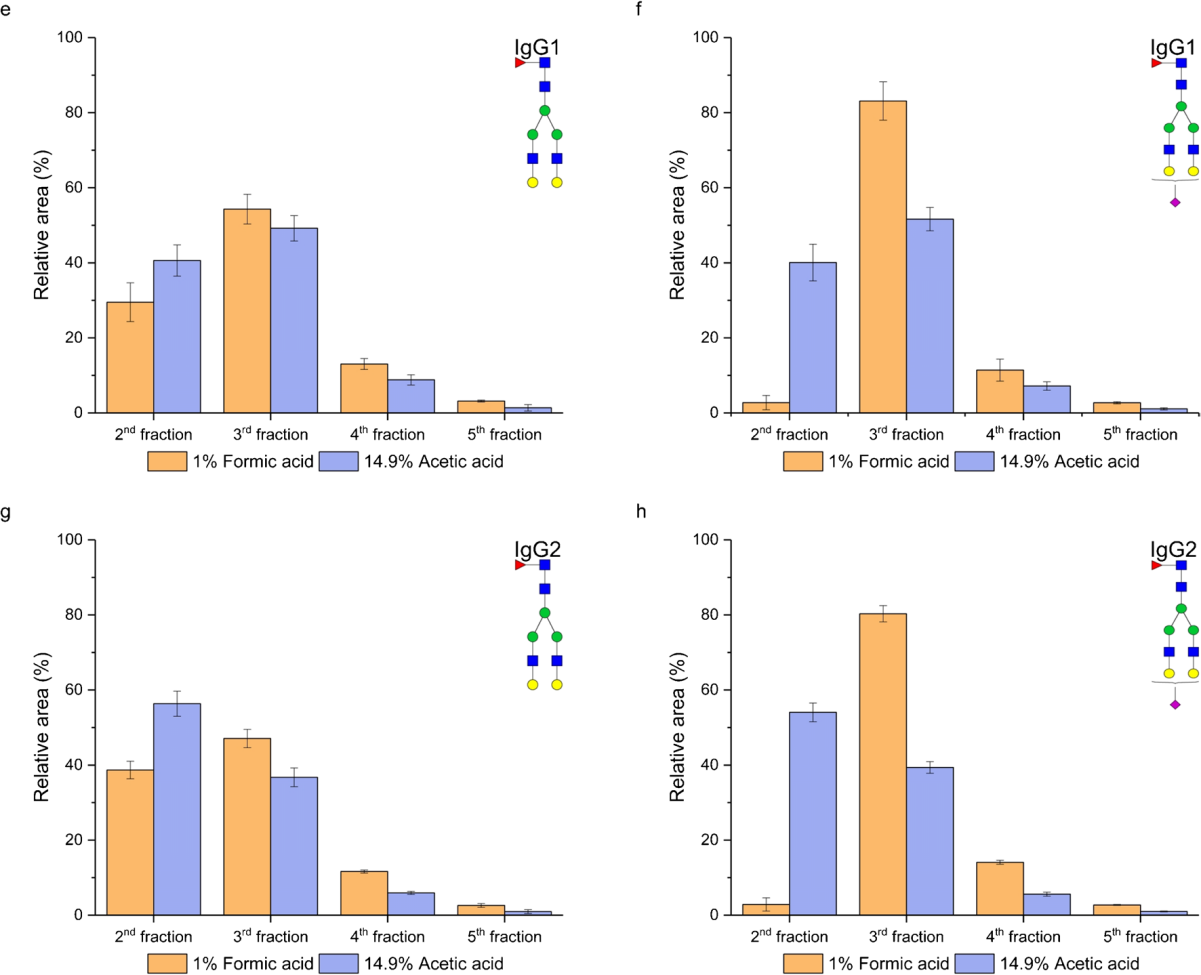
Effect of the acidifier type (formic and acetic acid) in the elution solvent for the lower (**a**–**d**) and higher (**e**–**h**) tested acid concentrations on glycopeptide enrichment efficiency

To assess the influence of elution solution acidifiers on enrichment efficiency, we substituted formic acid with acetic acid. Since acetic acid is a weaker acid than formic acid is (p*K*_a_ 4.75 vs p*K*_a_ 3.74), we adjusted its concentration to maintain comparable ionic strength and pH values with the different concentrations of formic acid (0.1% and 1%). The concentration of acetic acid was 1.4% (*I* = 2.16 mM, pH 2.68) and 14.9% (*I* = 7.37 mM, pH 2.17), respectively. In each case, the final concentration of acetonitrile was 5%. As can be seen in Fig. 6, both neutral (Fig. 6a, c, e, and g) and sialylated (Fig. 6b, d, f, and h) glycopeptides were eluted earlier when acetic acid was used in the elution solvent. The earlier elution was particularly notable for neutral IgG2 glycopeptides compared to IgG1 glycopeptides (Fig. 6c, g). Interestingly, for sialylated glycopeptides, we observed a greater effect of the acidifier type on enrichment efficiency. When 0.1% formic acid was used, none of these glycopeptides eluted in the second fraction (Fig. 6b, d). Conversely, with 1.4% acetic acid, 21% (Fig. 6b) of IgG1 and 39% (Fig. 6d) of IgG2 sialylated glycopeptides eluted in the second fraction. Similar trends were observed with higher tested concentrations of both formic acid and acetic acid (Fig. 6e–h). Our findings align with a recent study by Battellino [27], which demonstrated that substituting formic acid with acetic acid led to decreased peptide retention. This study suggested that acetic acid acts as a better hydrophilic ion pairing modifier due to its larger acetate counterion, which enhances steric hindrance, thereby increasing the hydrophobic interactions of terminal residues. In our case, the peptide backbones of both IgG1 and IgG2 contain two glutamic acids and a C-terminal carboxyl group, which are partly dissociated, as well as a positively charged arginine and a N-terminal amino group, both positively charged at pH 2.68 [22]. Moreover, the aminopropyl-modified stationary phase is positively charged at this pH. Based on this, we speculate that the acetate anion neutralizes the positive charge of both the stationary phase and the peptide backbone more effectively than the formate anion, resulting in earlier glycopeptide elution. This effect was more pronounced in partially dissociated sialylated glycoforms. These trends were observed for all studied neutral glycopeptides of IgG1 and IgG2 with both lower (Fig. S3 in Electronic Supplementary Material) and higher (Fig. S4 in Electronic Supplementary Material) tested acid concentrations.

### Effect of the acetonitrile amount in the elution solvent on glycopeptide enrichment

**Fig. 7:**
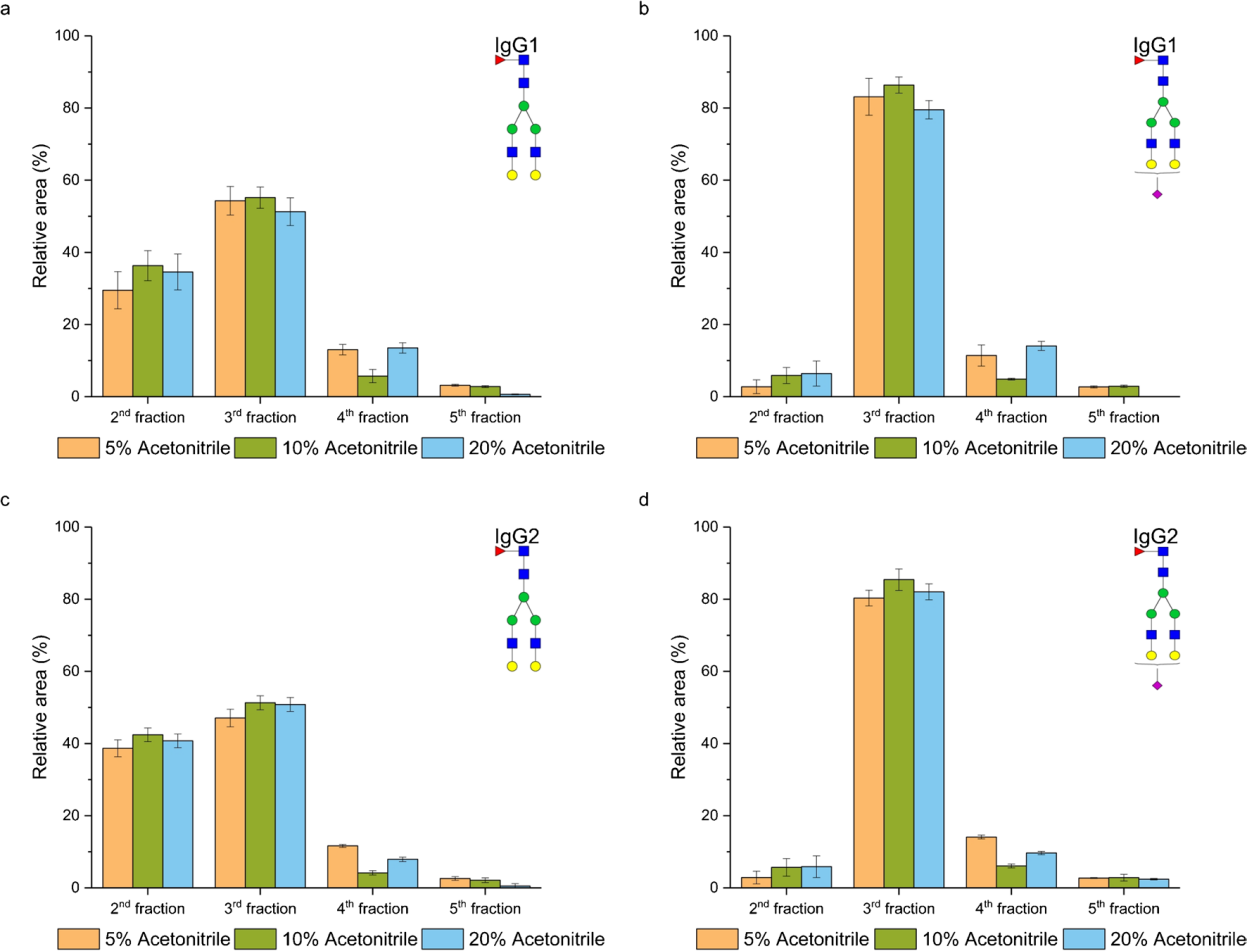
Effect of the acetonitrile concentration in the elution solvent on the enrichment efficiency of neutral glycopeptide of IgG1 (**a**) and IgG2 (**c**), and sialylated glycopeptide of IgG1 (**b**) and IgG2 (**d**)

Finally, we explored two different concentrations of acetonitrile for glycopeptide elution: specifically, concentrations of 10% and 20%, each with addition of 1% formic acid. In Fig. 7, it is seen that the enrichment efficiency of glycopeptides remained largely unaffected by the acetonitrile concentration in the elution solvent. We assume that this phenomenon may be attributed to the heightened elution strength of the solvent, primarily caused by a substantial amount of aqueous component. As a result, the concentration of acetonitrile within the investigated range in the elution solvent had no further impact on the enrichment efficiency. These observations were consistent across all studied glycopeptides, as demonstrated in Fig. S5 in Electronic Supplementary Material.

### Effect of the acetonitrile concentration during the conditioning and washing steps on glycopeptide enrichment

**Fig. 8:**
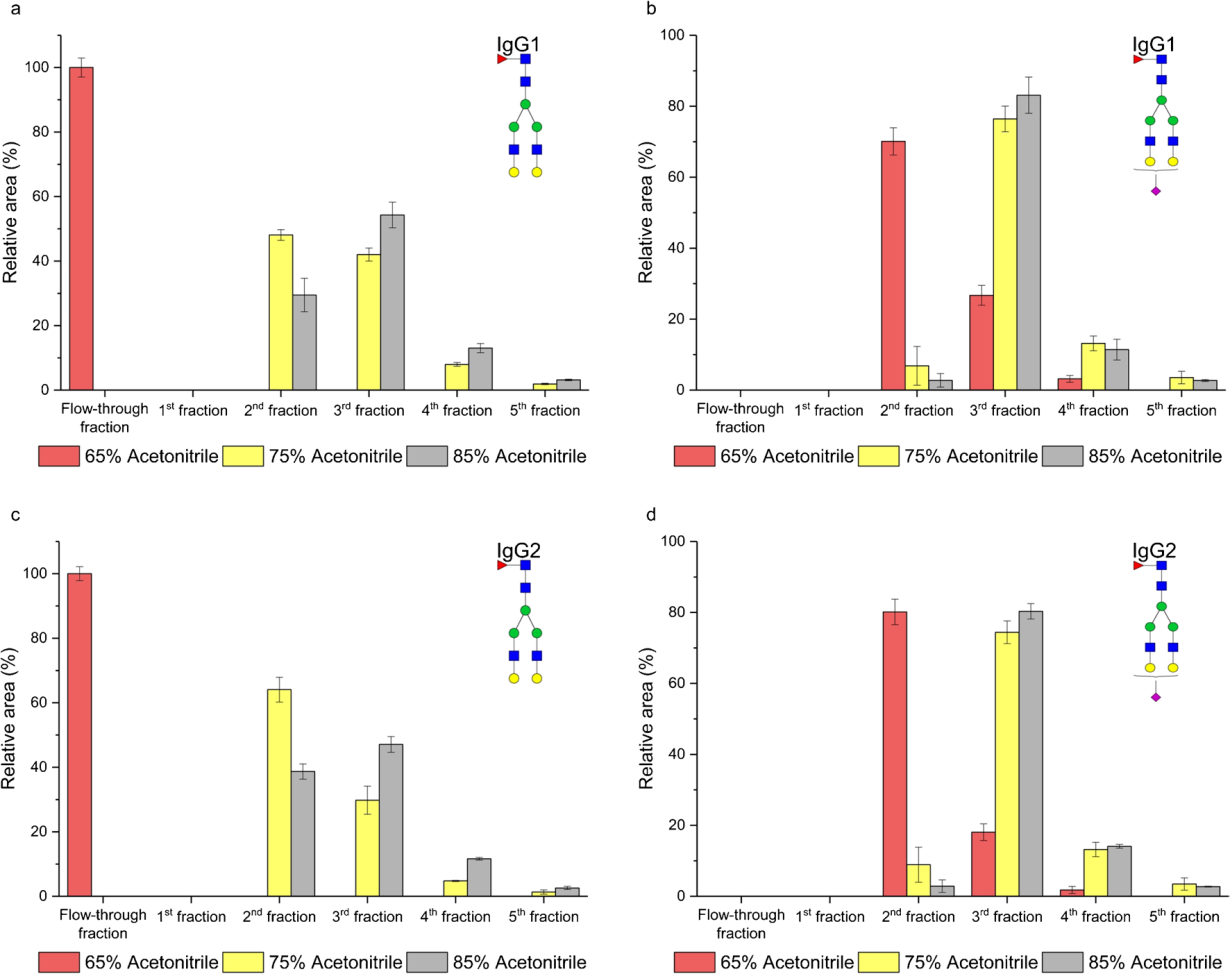
Effect of the acetonitrile concentration during the conditioning and washing steps on the enrichment efficiency of neutral glycopeptide of IgG1(**a**) and IgG2 (**c**), and sialylated glycopeptide of IgG1 (**b**) and IgG2 (**d**)

In the realm of HILIC, it is well-established that the concentration of acetonitrile plays a pivotal role in retaining polar analytes, since influencing the thickness of the adsorbed water layer on the polar stationary phase [28–32]. Based on these studies, we speculated whether the acetonitrile concentration used during the conditioning and washing steps could impact the retention of glycopeptides on the SPE column. To explore this, we tested three different concentrations: 85%, 75%, and 65% acetonitrile (each with the addition of 0.1% formic acid). The composition of the elution solvent remained unchanged, comprising 5% acetonitrile with addition of 1% of formic acid. In Fig. 8a and c, it is seen that decreasing the acetonitrile concentration in the solvent for the conditioning and washing the SPE column resulted in the earlier elution of the neutral glycopeptides. Furthermore, when a concentration of 65% acetonitrile was employed, these glycopeptides were exclusively found in the flow-through fraction, namely, in the washing fraction. This observation suggests either limited interaction of glycopeptides with the stationary phase or an inability to form an aqueous layer due to the composition of the mobile phase. With 75% acetonitrile, approximately 50% of neutral IgG1 glycopeptides (Fig. 8a) and 64% of IgG2 glycopeptides (Fig. 8b) eluted in the second fraction, whereas 80% acetonitrile resulted in higher retention of neutral glycopeptides, most of which eluted in the third fraction. This confirms our findings align with the notion that a high acetonitrile concentration results in stronger water adsorption on the stationary phase, elongating the retention time of polar analytes [24]. Regarding sialylated glycopeptides (Fig. 8b, d), we observed their successful retention even with 65% acetonitrile. More than 70% of IgG1 (Fig. 8b) and 80% of IgG2 (Fig. 8d) sialylated glycopeptides were detected in the second fraction. Interestingly, there was no significant difference in the enrichment efficiency of sialylated glycopeptides when 75% or 85% acetonitrile was used, suggesting the important role of ionic interactions as an additional retention mechanism between partly dissociated sialic acid and the positively charged stationary phase of the SPE column. The results obtained under these conditions for other studied glycopeptides provided similar trends and are depicted in Fig. S6 in Electronic Supplementary Material.

### Enrichment of the tryptic digest of human plasma

**Fig. 9:**
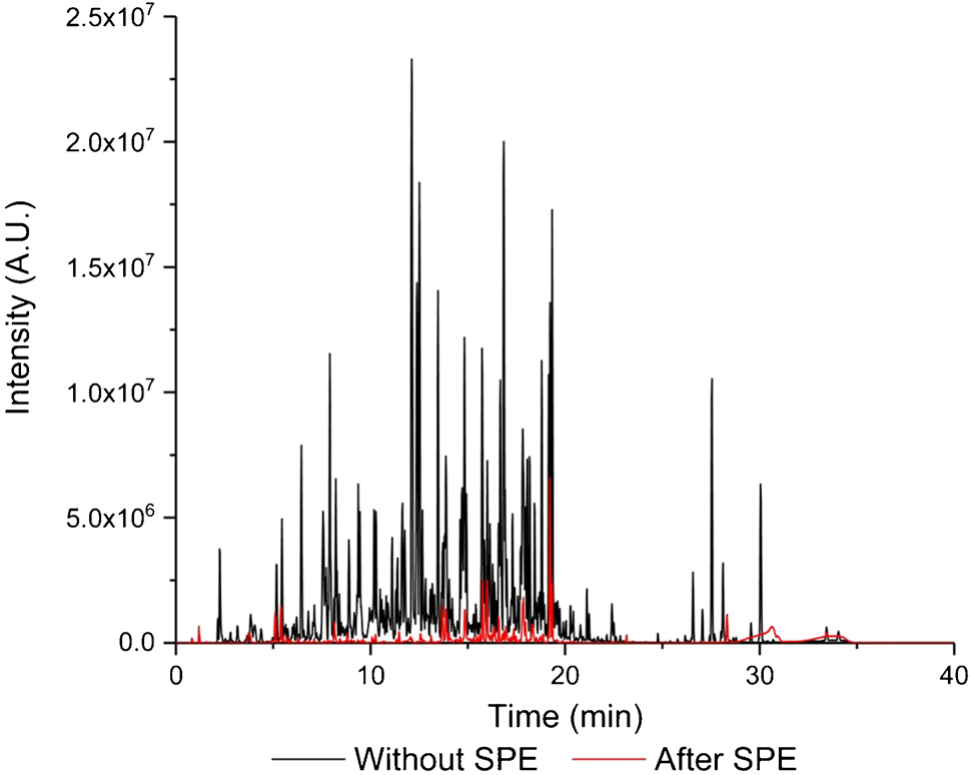
The base peak chromatograms of the trypsin-digested human plasma sample before SPE (black line) and after (red line) SPE treatment. The enrichment was carried out following the procedure outlined in the “Sample preparation and enrichment” section, with the five collected fractions being mixed, evaporated, and reconstituted in 100 µL of the elution solvent (5% acetonitrile with addition of 1% formic acid)

To assess the applicability of glycopeptide enrichment on the aminopropyl-modified SPE column for real samples, human plasma was digested based on a previous work published by Sanda and Goldman with minor modification [33]. Briefly, blood samples were collected using EDTA Vacutainer tubes (BD Diagnostics, Franklin Lakes, NJ), and plasma was separated within 60 min after collection and then immediately aliquoted and frozen at − 80 °C. For the digestion, 50 µL of plasma was used, which was first diluted in 1:20 with 50 mM sodium bicarbonate and processed as described in section “Sample preparation and enrichment.” Figure 9 presents the base peak chromatogram of the digested sample before (depicted by the black line) and after (depicted by the red line) the SPE pretreatment. It is evident that following the SPE treatment, the intensity of the later eluting components in the digest notably decreased, leading to an overall reduction in sample complexity.

**Fig. 10:**
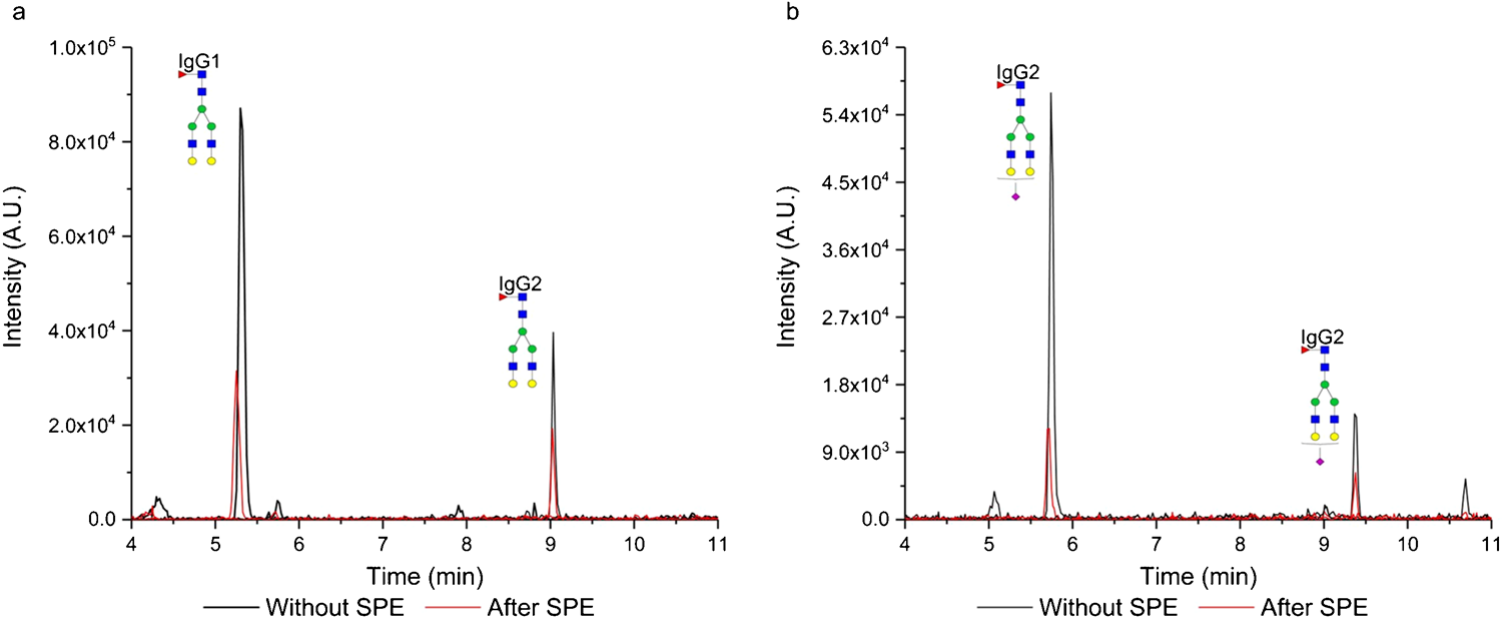
Comparison of the neutral (**a**) and sialylated (**b**) glycopeptide abundance before (black line) and after (red line) the enrichment step. The enrichment was carried out following the procedure outlined in the “Sample preparation and enrichment” section, with the five collected fractions being mixed, evaporated, and reconstituted in 100 µL of the elution solvent (5% acetonitrile with addition of 1% formic acid)

As IgG is the most abundant antibody [34], we were able to detect all the glycopeptides of IgG1 and IgG2 listed in Table 1. Upon comparing the absolute abundance of neutral digalactosylated (Fig. 10a) and digalactosylated-sialylated (Fig. 10b) glycopeptides of IgG1 and IgG2 before and after SPE pretreatment, we observed a decrease in their abundance. Specifically, the absolute intensity of the neutral glycopeptide of IgG1 decreased by a factor of 2.8, and in the case of IgG2, it decreased by a factor of 2.1. For the sialylated glycopeptides, these values were 4.1 (IgG1) and 3.0 (IgG2), respectively. Similar decreases in absolute abundance were observed for all the neutral and sialylated glycopeptides (Fig. S7 in Electronic Supplementary Material). Despite this reduction in absolute intensity, there was a substantial enhancement in the signal-to-noise ratio for all the analyzed glycopeptides, improving approximately twofold to fourfold.

**Fig. 11:**
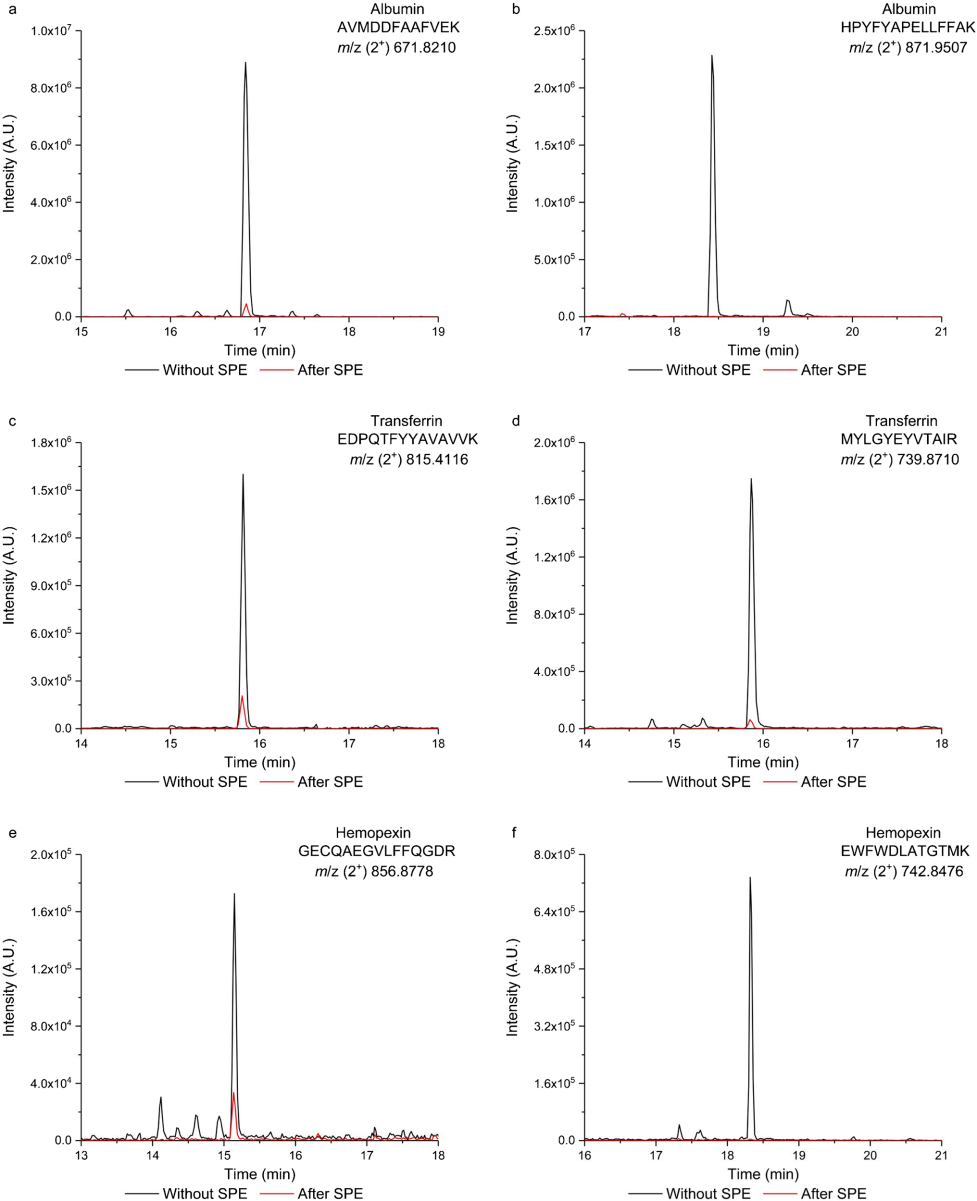
The extracted ion chromatograms of six highly abundant peptides of human plasma, i.e., albumin (**a**, **b**), transferrin (**c**, **d**), and hemopexin (**e**, **f**), before (black line) and after (red line) the enrichment on the aminopropyl-modified sorbent

We also investigated the relative abundance of highly concentrated peptides of the human plasma before and after the enrichment. Following the approach outlined in Hortin’s publication [35], we selected two abundant peptides from albumin (Fig. 11a, b) transferrin (Fig. 11c, d), and hemopexin (Fig. 11e, f). The extracted ion chromatograms clearly indicate a significant decrease in peptide abundance post-enrichment. Specifically, the intensity of the albumin peptide in Fig. 10a decreased by a factor of 26, while the other chosen peptide (Fig. 11b) was undetectable after the enrichment. For transferrin peptides, the intensities decreased by 7.7-fold (Fig. 11c) and 29-fold (Fig. 11d), and for hemopexin peptides, the decrease was 4.8-fold (Fig. 11e), while another studied peptide disappeared entirely post-enrichment (Fig. 11f). We attribute these variations in peptide abundance reduction to their differing polarities, affecting their interaction with the polar stationary phase.

Overall, the SPE enrichment process resulted in reduced background noise, enhancing the analysis of low-abundance glycopeptides in human plasma. In Table 2, we summarize the absolute areas of the studied glycopeptides and peptides before and after the enrichment, the ratio of the absolute areas (absolute area before the enrichment/absolute area after the enrichment), and the decrease in the area after the enrichment procedure (100 − (100*(absolute area after the enrichment/absolute area before the enrichment))). As no additional glycopeptides were detected beyond the fifth fraction, we hypothesize that the reduced intensity of the studied glycopeptides may be attributed to the matrix effect of human plasma. Human plasma comprises a complex matrix containing various components such as proteins, lipids, salts, and ions, all of which can influence the ionization efficiency of different analytes in the electrospray, either by suppressing or enhancing it [36]. After SPE enrichment, the composition of the sample matrix underwent changes, potentially influencing the ionization efficiency of the targeted analytes. In our case, it is likely that certain compounds originally supporting glycopeptide ionization efficiency were removed from the matrix during the SPE enrichment process. Consequently, we observed a reduction in the intensities of the studied glycopeptides. Overall, the SPE enrichment process resulted in reduced background noise, enhancing the analysis of low-abundance glycopeptides in human plasma.

**Table 2:** The summarized data of the studied glycopeptides and peptides of human plasma before and after the enrichment procedure

	Area before SPE (mAU min^−1^)	Area after SPE (mAU min^−1^)	Ratio (area before SPE/area after SPE)	Decrease in area (%)
IgG1				
FA2	517221	210946	2.4	59
FA2G1	978233	364247	2.7	63
FA2G2	422261	149636	2.8	65
FA2G1S1	56511	16107	3.5	71
FA2G2S1	206338	50233	4.1	75
IgG2				
FA2	424745	238500	1.8	44
FA2G1 (isomer 1)	164764	79389	2.1	52
FA2G1 (isomer 2)	236060	111751	2.1	52
FA2G2	108120	51300	2.1	52
FA2G1S1	39720	20927	1.9	47
FA2G2S1	50300	16729	3.0	66
Albumin				
AVMDDFAAFVEK	34540516	1334989	36.9	96
HPYFYAPELLFFAK	7264008	0	-	100
Transferrin				
MYLGYEYVTAIR	6215449	211545	29.4	96
EDPQTFYYAVAVVK	5077576	659012	7.7	87
Hemopexin				
GECQAEGVLFFQGDR	575456	119640	4.8	79
EWFWDLATGTMK	2096794	0	-	100

## Conclusion

In summary, in this paper, we present a study dealing with different experimental conditions for the enrichment of human IgG glycopeptides by SPE on an aminopropyl-modified stationary phase in HILIC mode. The glycopeptides of IgG1 exhibited a stronger interaction with the polar stationary phase compared to IgG2 glycopeptides due to the higher hydrophilicity of the peptide backbone. Additionally, ionic interactions were observed between sialylated glycopeptides and the aminopropyl-modified stationary phase of the SPE column, resulting in their later elution. Our study revealed that isopropanol proved to be the least effective organic solvent for enriching the studied glycopeptides, while acetonitrile demonstrated the highest efficiency for this purpose, with methanol falling between the other two tested solvents. Furthermore, higher concentrations of formic acid in the elution solvent suppressed ionic interactions, weakening the interaction of glycopeptides with the SPE column, especially noticeable for sialylated glycopeptides. Substituting formic acid with acetic acid led to the earlier elution of more glycopeptides. The concentration of acetonitrile used for conditioning and washing solutions significantly influenced the glycopeptide enrichment by affecting the thickness of the adsorbed water layer on the SPE stationary phase, thereby significantly impacting enrichment efficiency. Using 65% acetonitrile resulted in glycopeptides not being retained on the aminopropyl-modified SPE column and being detected in the flow-through fraction. Ultimately, it was demonstrated that the enrichment method was applicable to human plasma samples, resulting in a significant reduction in the abundances of non-glycosylated peptides. To the best of our knowledge, this study represents the first systematic investigation into the impact of the mobile phase on glycopeptide enrichment using an aminopropyl-modified SPE column in HILIC mode. Thus, these findings introduce a novel dimension to glycoproteomic studies by thoroughly investigating the influence of experimental condition on the enrichment efficiency. These results underscore the pivotal role of solvent composition and conditions in fine-tuning glycopeptide enrichment, thereby providing crucial insights for achieving robust analytical outcomes in glycoproteomic analyses.

### Supplementary Information

Below is the link to the electronic supplementary material.

Supplementary file1 (PDF 1.19 MB)
