# Supplementary material for: IgG glycopeptide enrichment using hydrophilic interaction chromatography-based solid-phase extraction on an aminopropyl column
Source: Anal Bioanal Chem. 2024 Feb 13;416(8):1867–81. doi: 10.1007/s00216-024-05187-y (PMC10901958; doi:10.1007/s00216-024-05187-y)
Supplement: Supplementary file 1 — Supplementary file1 (PDF 1.19 MB) [file 216_2024_5187_MOESM1_ESM.pdf]

IgG Glycopeptide Enrichment Using Hydrophilic Interaction Chromatography-based Solid-Phase Extraction on an Aminopropyl column

Supplementary information

Katarina Molnarova[1], Michaela Chobotova[1], Petr Kozlik[1,*]

[1] Department of Analytical Chemistry, Faculty of Science, Charles University, Prague, Czech Republic

Corresponding author:

RNDr. Petr Kozlik, Ph.D., Charles University, Faculty of Science, Department of Analytical Chemistry, Hlavova 8, 128 43 Prague 2, Czech Republic.

Tel.: +420 221 951 218.

E-mail address: kozlik@natur.cuni.cz

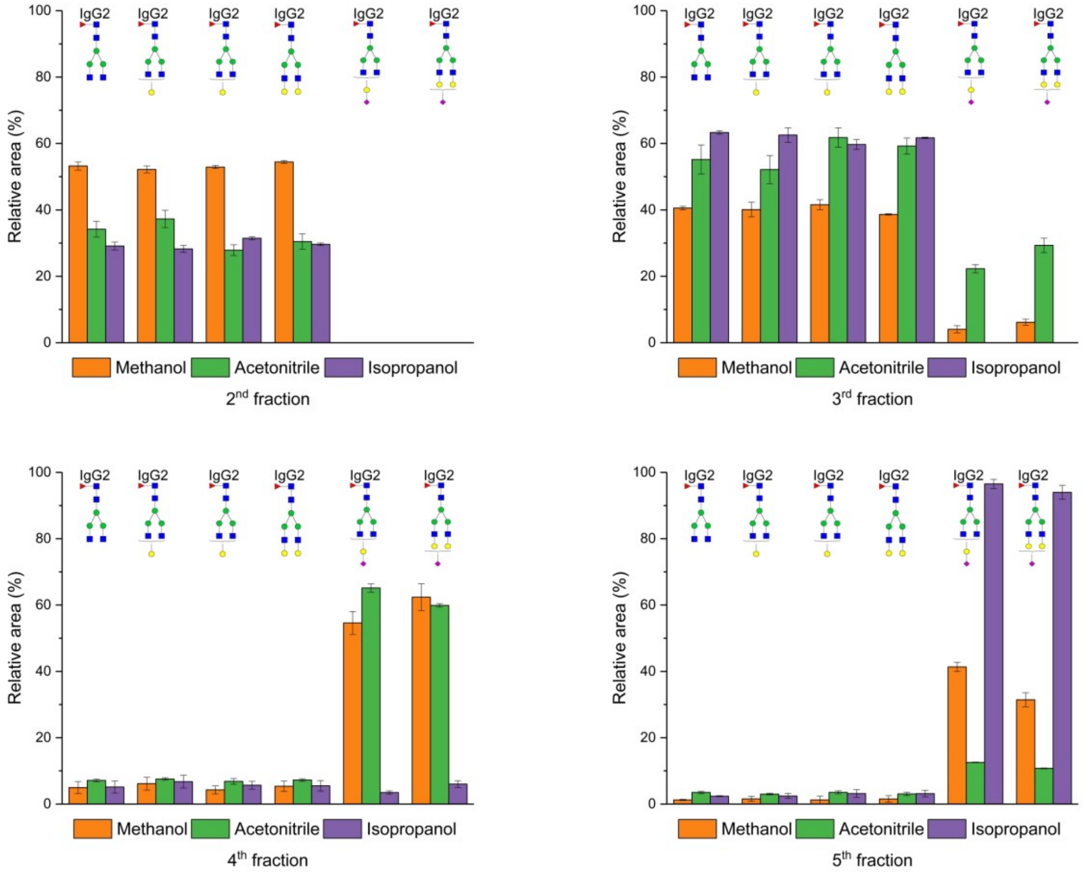

Figure S1 Effect of different organic modifiers of the elution solvent on the efficiency of IgG2 glycopeptide enrichment.

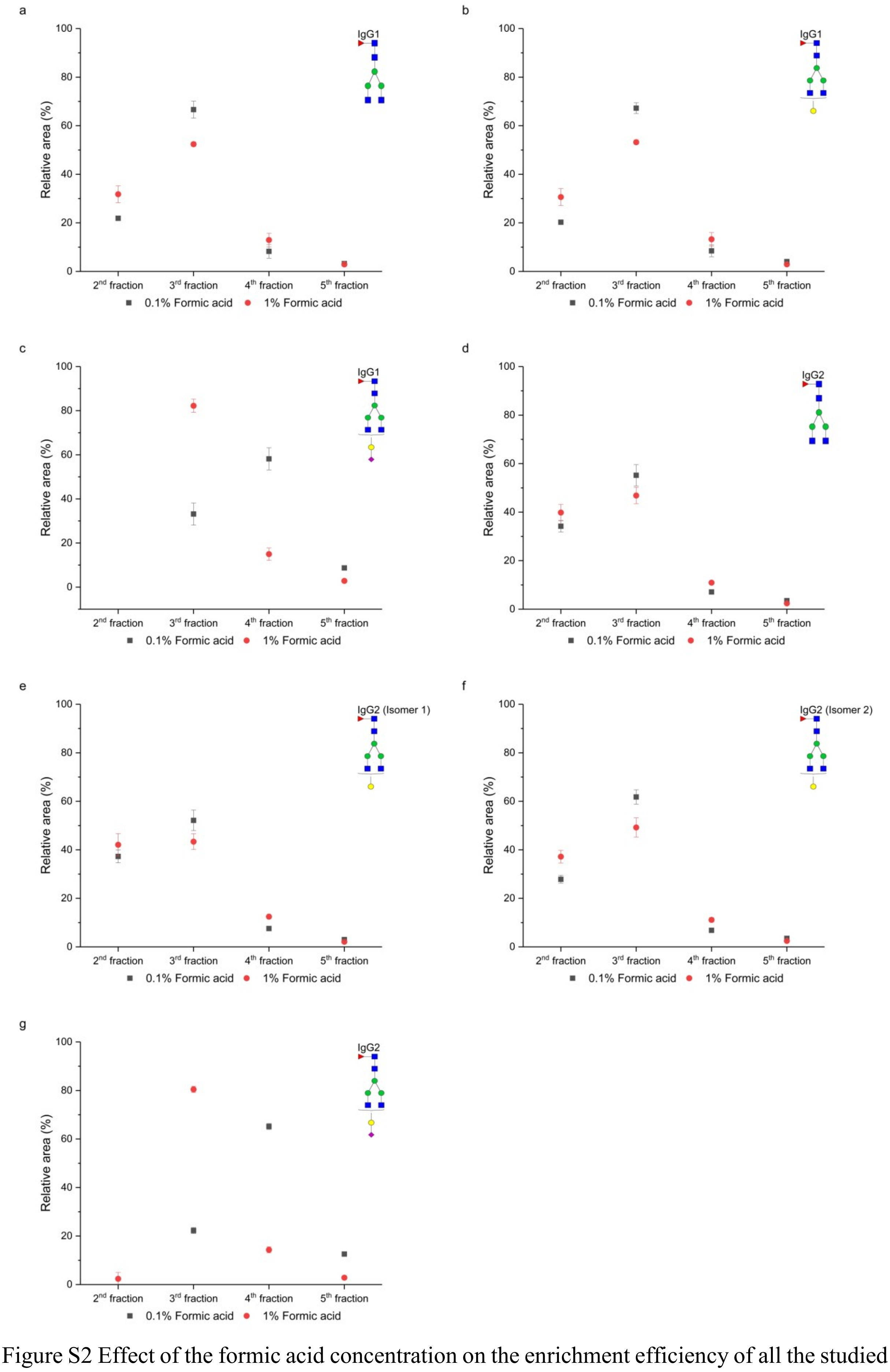

Figure S2 Effect of the formic acid concentration on the enrichment efficiency of all the studied glycopeptides of IgG1 (A-C) and IgG2 (D-G).

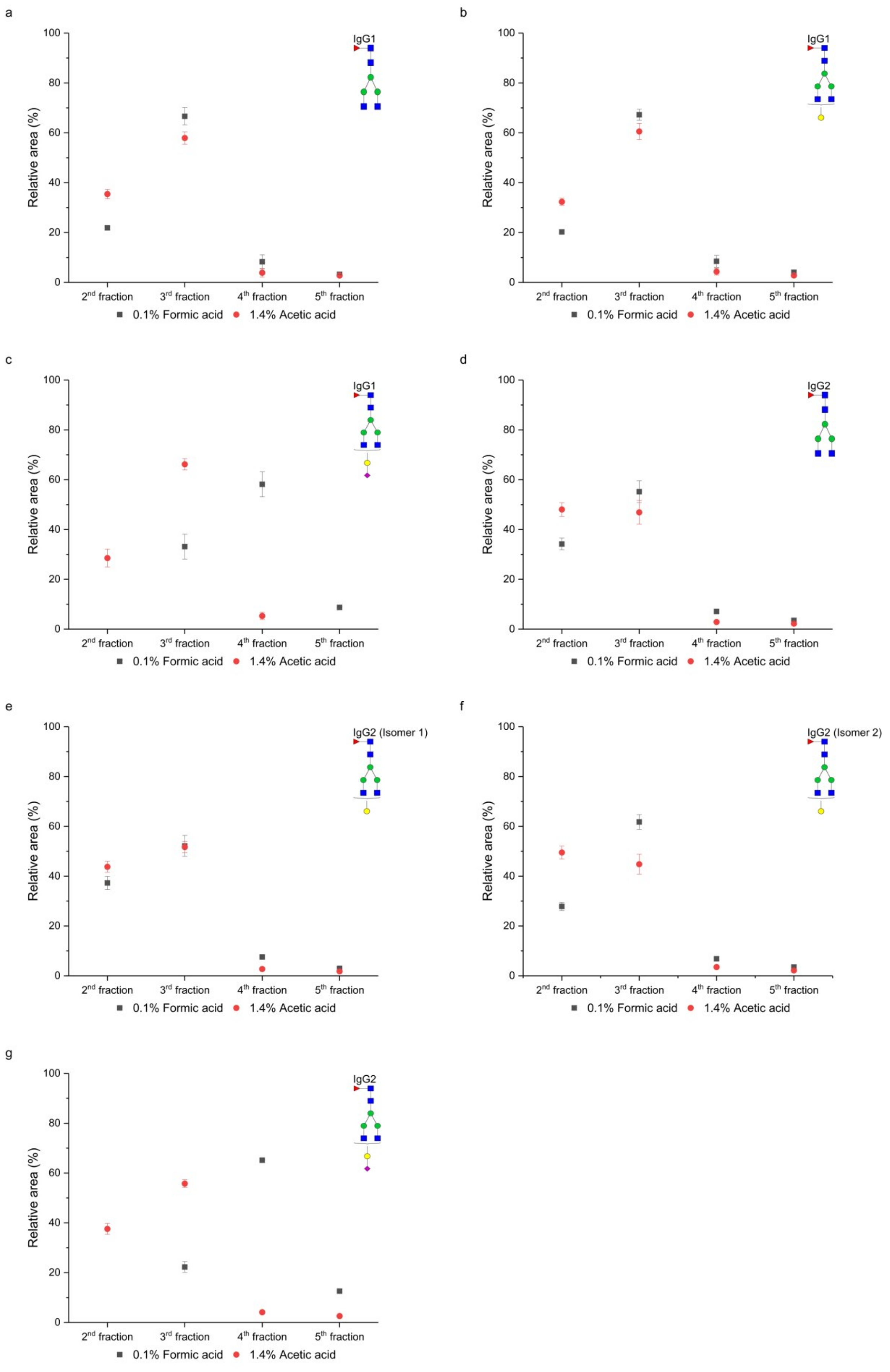

Figure S3 Effect of the acidifier type (formic and acetic acid) at low concentrations in the elution solvent on the enrichment efficiency of all the studied glycopeptides of IgG1 (A-C) and IgG2 (D-G).

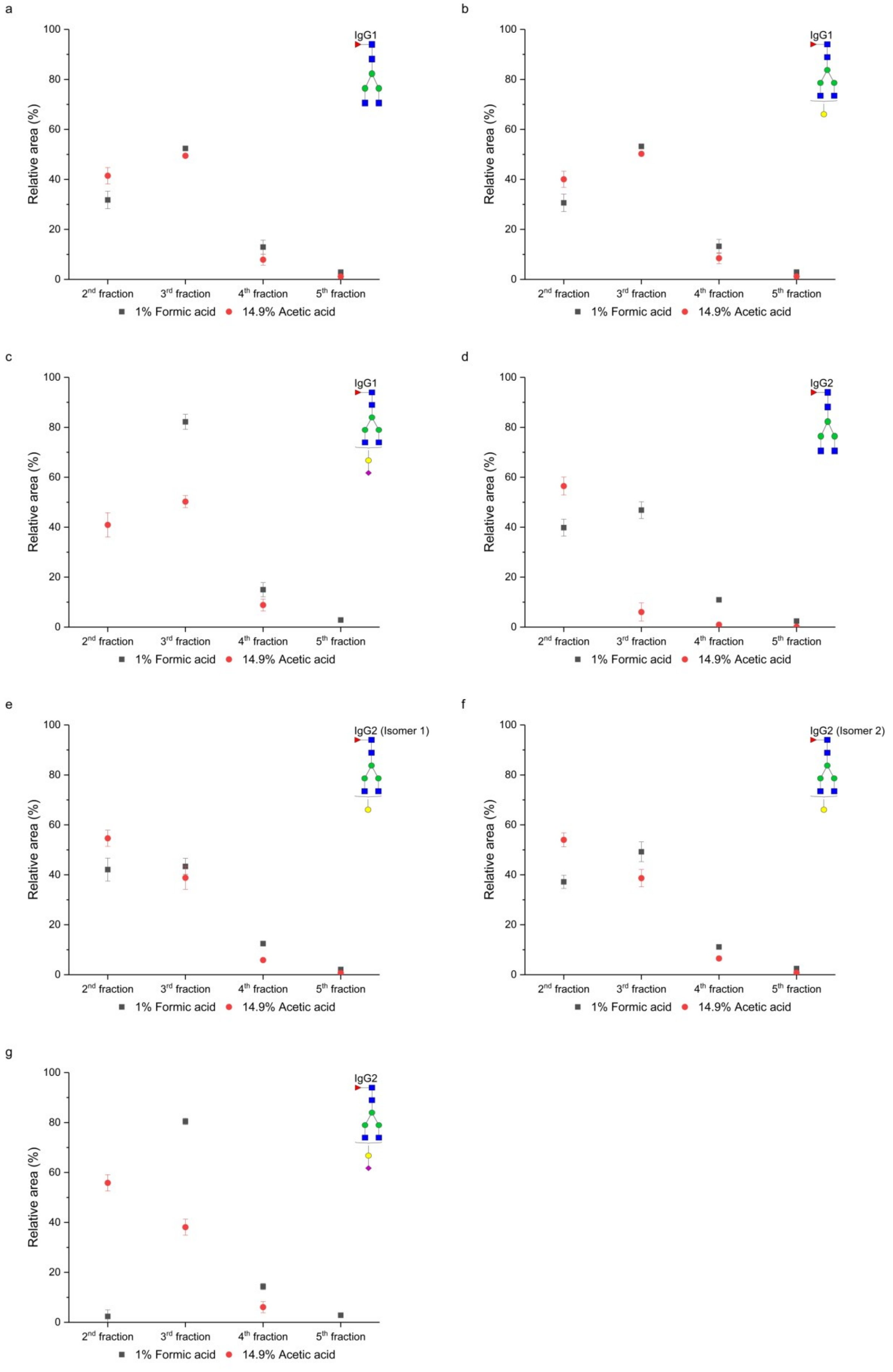

Figure S4 Effect of the acidifier type (formic and acetic acid) at high concentrations in the elution solvent on the enrichment efficiency of all the studied glycopeptides of IgG1 (A-C) and IgG2 (D-G).

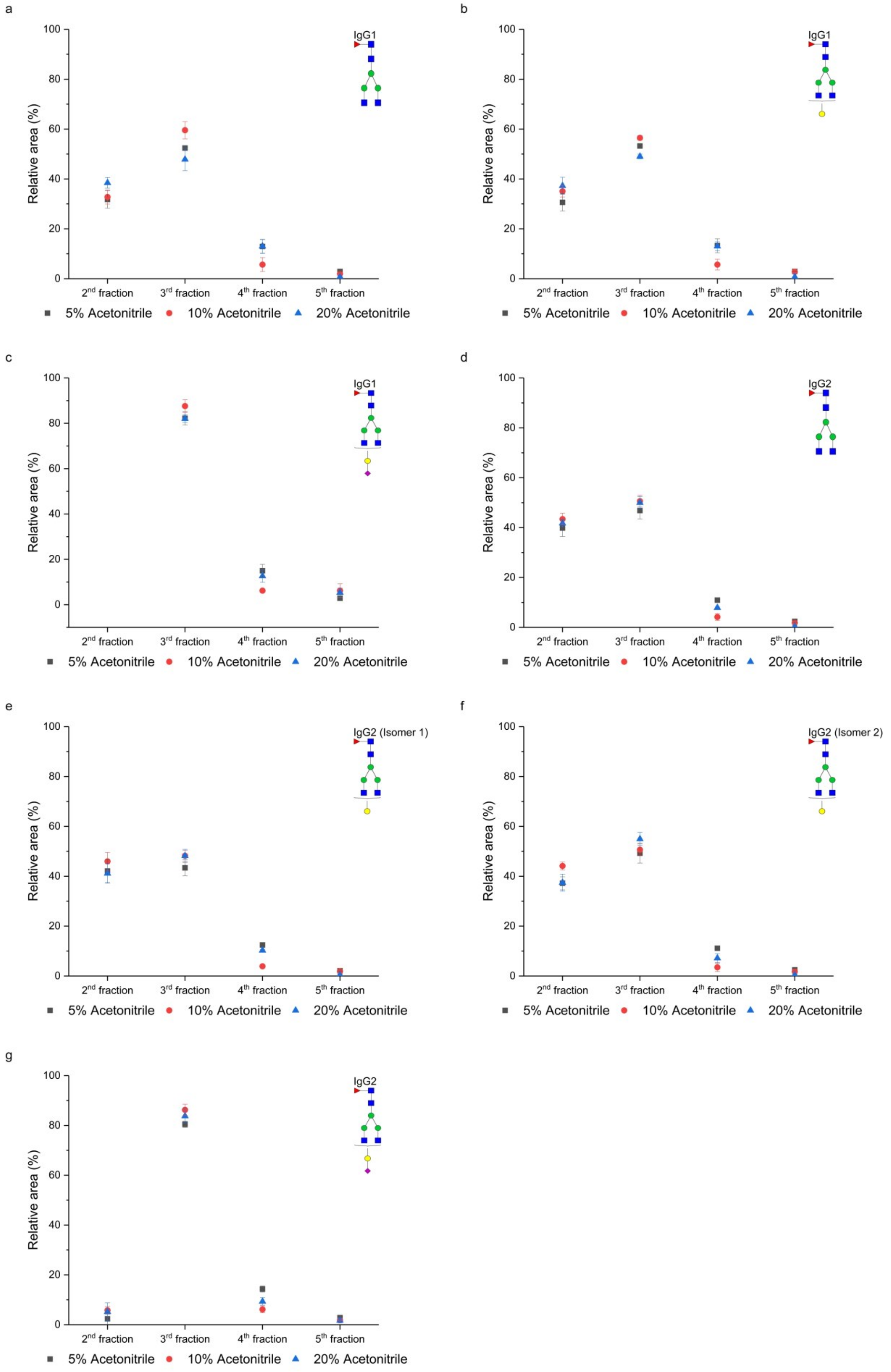

Figure S5 Effect of the acetonitrile concertation in the elution solvent on the enrichment efficiency of the glycopeptides of IgG1 (A-C) and IgG2 (D-G).

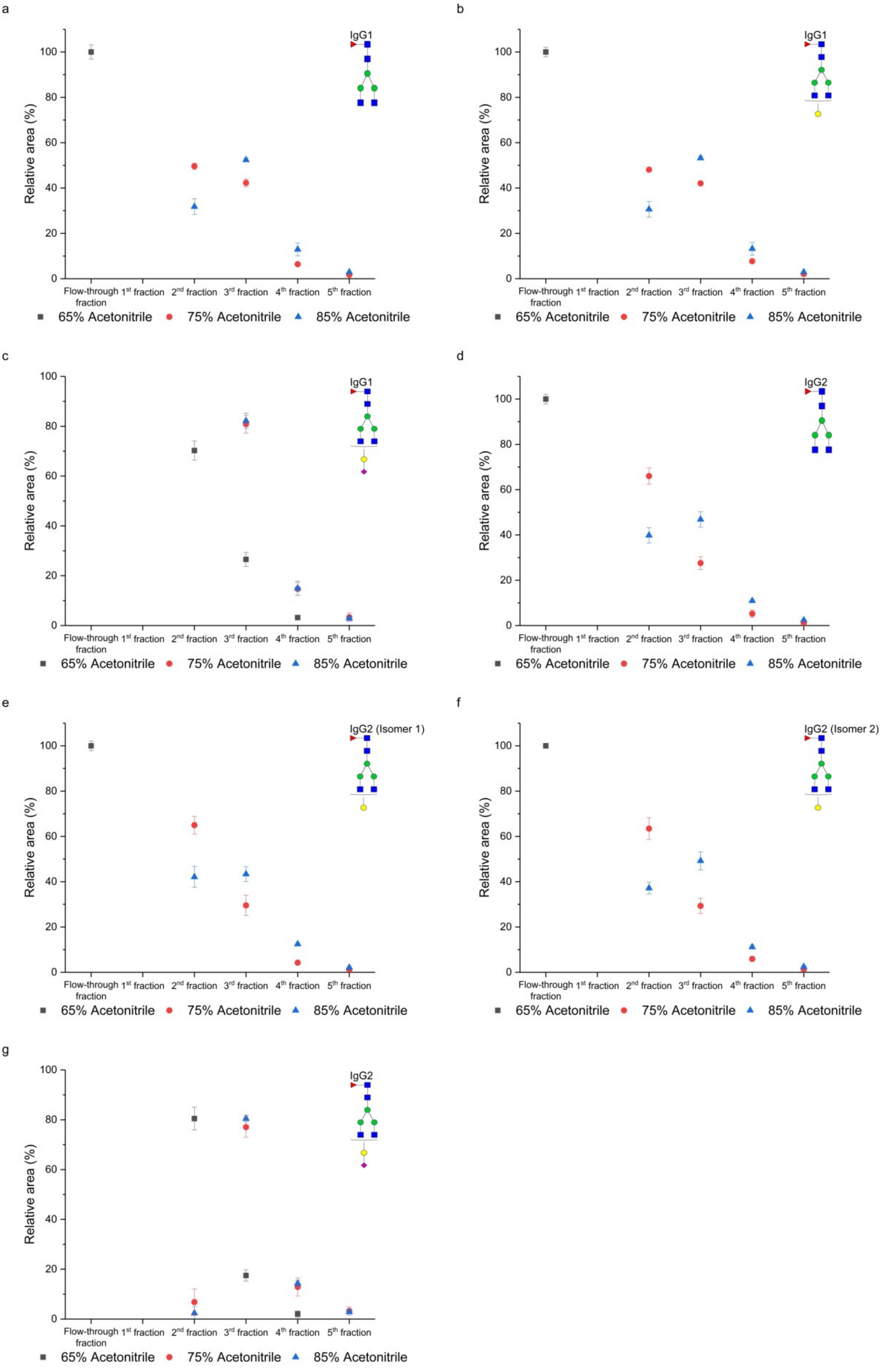

Figure S6 Effect of the acetonitrile concentration during the conditioning and washing steps on the enrichment efficiency of the glycopeptides of IgG1 (A-C) and IgG2 (D-G).

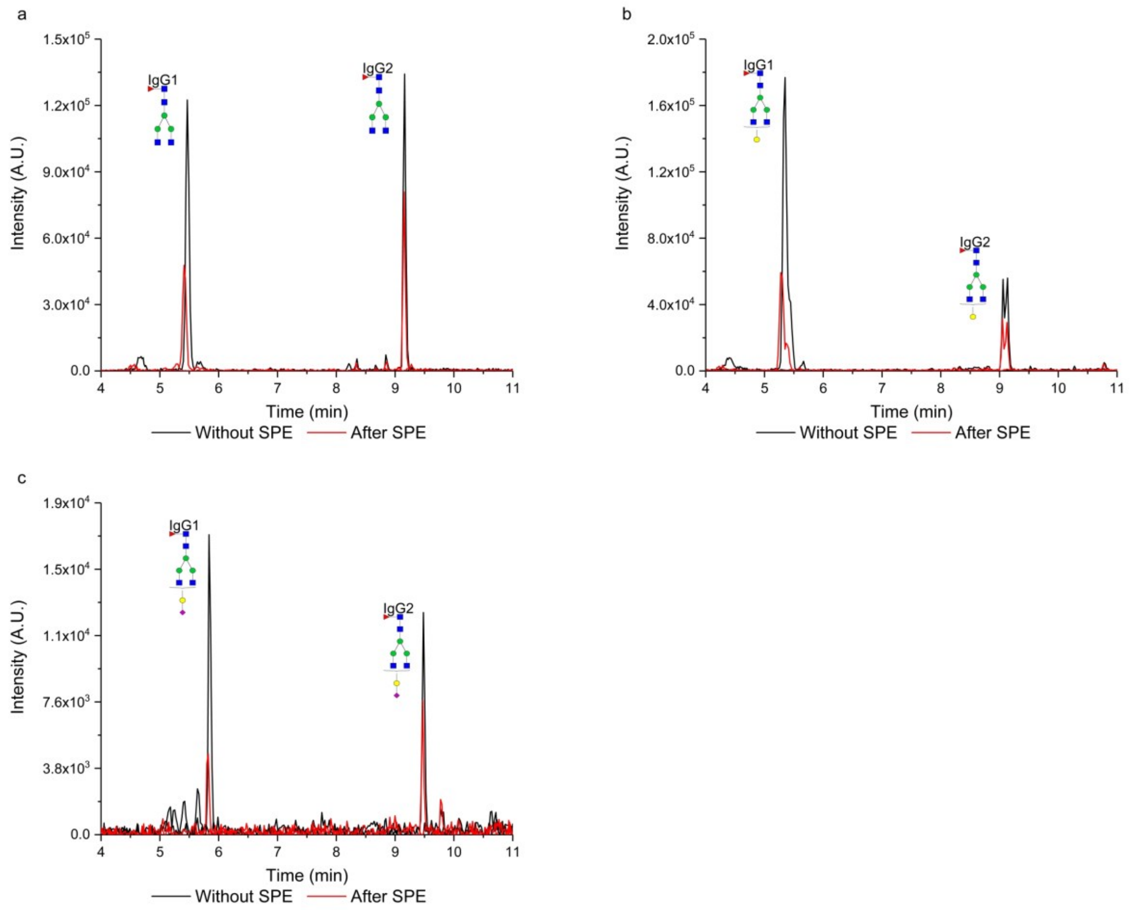

Figure S7 Comparison of the glycopeptides abundance before (black line) and after (red line) the enrichment step. The enrichment was carried out following the procedure outlined in the "Sample Preparation and Enrichment" section, with the five collected fractions being mixed, evaporated, and reconstituted in 100 µL of the elution solvent (5% acetonitrile with addition of 1% formic acid).
